# Organization of the epileptogenic zone and signal analysis at seizure onset in patients with drug‐resistant epilepsy due to focal cortical dysplasia with mTOR pathway gene mutations—An SEEG study

**DOI:** 10.1002/epi4.12810

**Published:** 2023-08-22

**Authors:** Irina Oane, Andrei Barborica, Andrei Daneasa, Mihai Dragos Maliia, Jean Ciurea, Sergiu Stoica, Aurelia Dabu, Flavius Bratu, Camelia Lentoiu, Ioana Mindruta

**Affiliations:** ^1^ Epilepsy Monitoring Unit University Emergency Hospital Bucharest Bucharest Romania; ^2^ Physics Department University of Bucharest Bucharest Romania; ^3^ Epilepsy Monitoring Unit University Hospital Rennes Rennes France; ^4^ Neurosurgery Department “Bagdasar‐Arseni” Emergency Hospital Bucharest Romania; ^5^ Neurosurgery Department Brain Institute, Monza Hospital Bucharest Romania; ^6^ Neurosurgery Department University Emergency Hospital Bucharest Bucharest Romania; ^7^ Neurology Department, Faculty of Medicine University of Medicine and Pharmacy Carol Davila Bucharest Bucharest Romania

**Keywords:** epilepsy surgery, focal cortical dysplasia, mTOR mutation, SEEG

## Abstract

Epilepsy surgery in genetic drug‐resistant epilepsy is a debated subject as more histological and molecular data are available. We retrospectively collected data from focal drug‐resistant epilepsy patients that underwent stereoelectroencephalography (SEEG) invasive recordings. Patients with nonlesional brain imaging or in whom a first epilepsy surgery failed to control seizures were selected. We computed and displayed the intracranial ictal onset activity pattern on structural imaging. Patients underwent epilepsy gene panel testing, next generation sequencing—NGS. Of 113 patients, 13 underwent genetic testing, and in 6 patients, a mechanistic target of rapamycin pathway gene germline mutation (mTOR) was identified. Brain imaging was nonlesional except for one patient in whom two abnormalities suggestive of focal cortical dysplasia (FCD) were found. Patients underwent tailored brain surgery based on SEEG data, tissue analysis revealed FCD and postsurgical outcome was favorable. Our findings are similar to previous case series suggesting that epilepsy surgery can be a treatment option in patients with mTOR pathway mutation. In patients with mTOR pathway mutation, the postsurgical outcome is favorable if complete resection of the epileptogenic zone is performed. Electrophysiological seizure onset patterns in FCDs associated with mTOR pathway mutations display low‐voltage fast activity as previously described.

## 1 INTRODUCTION

Focal cortical dysplasia (FCD) is the most common etiology among patients with focal structural drug‐resistant epilepsy and almost 70% of cases that undergo epilepsy surgery, particularly in FCDII achieve seizure freedom at 5 years.^1^ In the last decade, new knowledge has become available regarding the role of somatic or germline pathogenic gene variants in the etiology of FCD, and an update of FCD classification has been proposed.^2^ There is an increasing evidence that FCDII is associated with hyperactivation of the mechanistic target of rapamycin (mTOR) pathway.^3^ Brain somatic mutations in the PI3K‐PTEN‐AKT3‐TSC pathway and germline mutations in GATOR genes plus double‐hit somatic mutations in DEPDC5 have been described in cases of malformation of cortical development.^3^ Until recently, patients with pathogenic gene variants have been discarded from epilepsy surgery mostly because of lack of data related to postsurgical follow‐up in these cases. However, it has been previously suggested that surgical outcome in patients with epilepsy due to mTOR pathway gene mutations is much more successful than in those related to genes involved in channel functions and synaptic transmission.^4^

In this paper, we report six patients with drug‐resistant epilepsy explored by stereoelectroencephalography (SEEG), addressing the issue of whether there is a focal organization of the epileptogenic zone in cases without a clear‐cut magnetic resonance imaging (MRI) lesion, genetic testing positive for an mTOR mutation and pathology concluding a diagnosis of FCD II. We also characterized the ictal discharge at seizure onset using signal analysis tools.

## 2 MATERIALS AND METHODS

We have retrospectively collected clinical, electrophysiological, imaging, and genetic data from patients with focal drug‐resistant epilepsy that underwent SEEG at the University Emergency Hospital between 2012 and 2022. We included patients that had nonlesional brain imaging (MRI) or in whom, a first epilepsy surgery failed to control seizures.

Patients underwent presurgical evaluation consisting in long‐term video‐electroencephalography (video‐EEG), brain MRI, interictal 18Fluorodeoxyglucose‐positron emission tomography (FDG‐PET), and neuropsychological assessment. Invasive intracranial recordings were considered necessary to delineate the seizure onset zone (SOZ) and to map the functional cortex using direct brain electrical stimulation.^5^ SEEG exploration was performed using intracranial electrodes with 12–18 contacts, 2 mm contact length, 1.5 mm contact spacing, and 0.8 mm diameter (Dixi Medical). Long‐term SEEG recordings (average of 10 days) were performed using a 64 Wireless or 128‐channel XLTek Quantum system (Natus). SEEG traces were evaluated by two epileptologists to delineate the SOZ. To better visualize the electrical seizure onset pattern,^6^, ^7^ either instantaneous activations in the gamma band (30–80 Hz)^8^ or the instantaneous weighted power ratio (IWPR) were computed and plotted on the patients specific structural MRI.^9^ The IWPR, derived from the energy ratio^10^ combined with instantaneous activations based on Hilbert transform,^8^ quantifies the involvement of each structure explored by SEEG electrodes to the seizure onset by combining low‐voltage fast activity and slow activity. Patients consented, and the presurgical investigation was performed under the Ethical Committee approval No2621/03.02.2012. Patients were recommended gene panel, next generation sequencing—NGS (Invitae epilepsy panel) before invasive recordings or retrospectively but before a second resective surgery or a second SEEG exploration.

## 3 RESULTS

Of 113 patients that underwent SEEG recordings, 45 patients fulfilled the inclusion criteria, 13 patients consented to genetic testing, and in six, we have found a germline gene mutation related to mTOR pathway signaling (Table 1). In five patients, the mutation was found after the first invasive recordings (patient 1–5), and in one patient, (patient 6) genetic testing results were available before SEEG.

**TABLE 1:** Demographic, clinical, electrophysiological and genetic data, family history, imaging, and pathology data related to the cases presented.

Patient	Gender	Age at SEEG (years)	Age at epilepsy onset (years)	Seizure semiology	Scalp EEG findings	Brain MRI	SEEG exploration results (epileptogenic zone)	Pathology	Mutation			Family history of epilepsy	Surgical outcome	Follow‐up (months)	ASM
									Gene	Variant	ACMG class				
1	M	42	13	Sensation of contrast‐enhanced, hyperclear‐vision, loss of consciousness and genital automatisms	Bilateral frontal predominantly left‐sided, mesial and dorsolateral region	Nonlesional	Left‐frontal pole, orbitofrontal, anterior cingulate cortex, dorsomedial prefrontal, middle cingulate cortex, presupplementary motor area, and superior frontal	FCD IIA	SZT2	c.3637C>T (p.Gln1213*)	Pathogenic	No	Engel IA	92	removed
2	M	7	6	Smiling grimace followed by hypermotor behavior	Bilateral frontal regions, mostly the left parasagittal	Nonlesional	Left pregenual anterior cingulate cortex	FCD IIA	DEPDC5	c.3205C>T (p.Gln1069*)	Pathogenic	No	Engel IIA	63	Valproic Acid
3	M	18	6	Strange sensation in the left hemi‐body particularly the leg followed by bilateral tonic contraction	Bilateral frontal‐central predominantly over the right‐midline	Nonlesional	Right middle cingulate cortex, supplementary motor area, motor area (paracentral lobule)	FCD IIB	NPRL3	Partial deletion (exon 12)	Pathogenic	Brother	Engel IIIA	46	Oxcarbazepine, Levetiracetam
4	M	20	1	Fear and a strange sensation over his left hemi‐body particularly in the lower limb followed by hypermotor behavior, grasping, face‐flushing	Midline and frontal‐central abnormalities	Nonlesional	Right middle cingulate cortex, supplementary motor area, paracentral lobule, anterior cingulate cortex and anterior insula	FCD IIA	NPRL3	c.562C>T (p.Gln188*)	Pathogenic	No	Engel IVA	66	Valproic Acid, Lamotrigine
5	M	20	2	Limbs paresthesia (predominantly the right side), eye blinking, laughter, hypersalivation and throat contraction, sometimes followed by tonic–clonic bilateralization	Left frontal and midline	Nonlesional	Left middle cingulate cortex and presupplementary motor area	FCD IIA	DEPDC5	c.1191T>A (p.Tyr397*)	Pathogenic	No	Engel IA	56	Oxcarbazepine
6	M	18	10	Left‐side tonic eye‐version followed by bilateral tonic–clonic contractions	Right dorsolateral frontal‐central	Two lesions (FCD II)	Right frontal eye field	FCD IIB	DEPDC5	c.4398G>A (p.Trp1466*)	Pathogenic	No	Engel IA	20	removed

Abbreviations: FCD, focal cortical dysplasia; MRI, magnetic resonance imaging; SEEG, stereoelectroencephalography.

**FIGURE 1:**
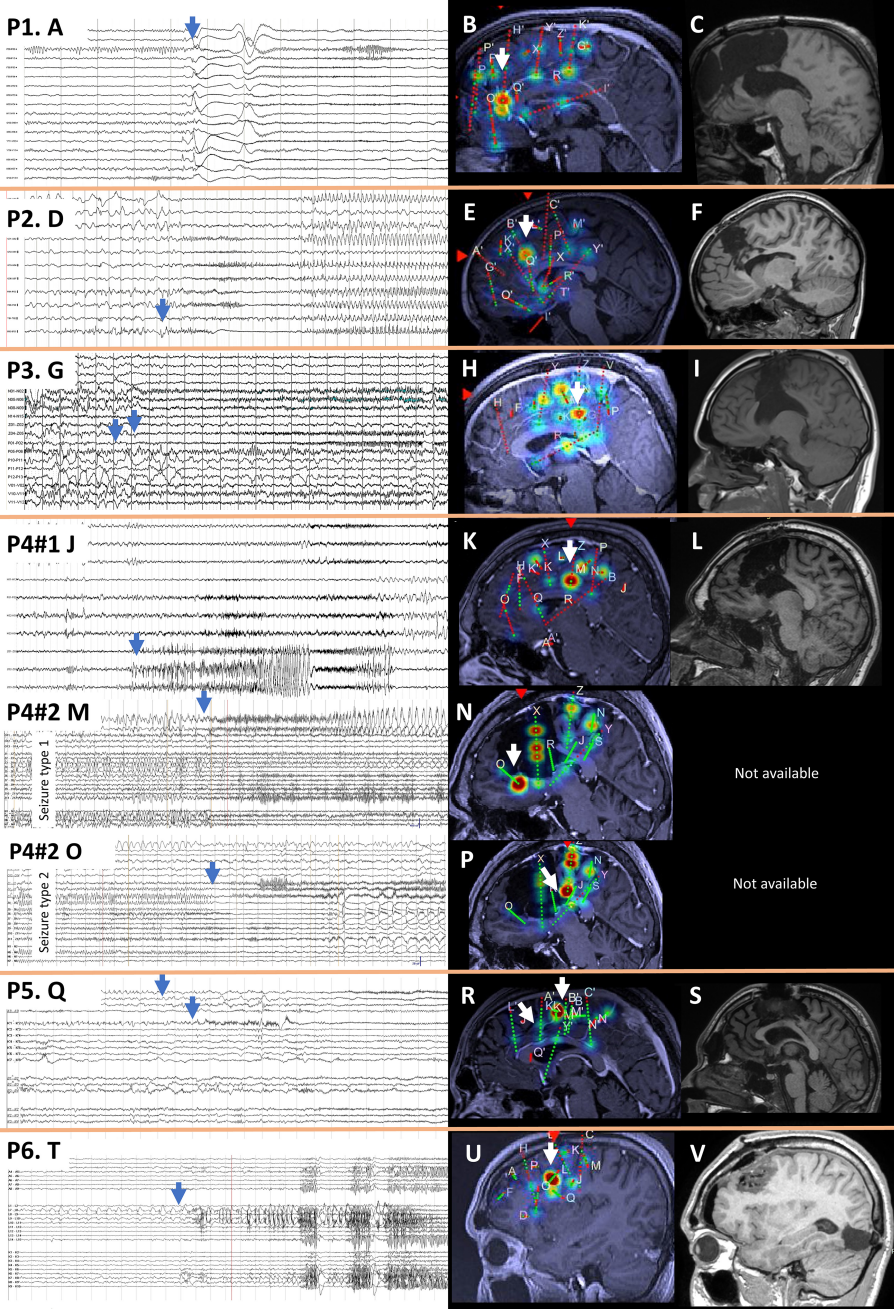
Intracranial electrophysiology, ictal activity maps in the gamma band (patients 1–3) or instantaneous weighted power ratio (IWPR) (patients 4–6) and postsurgical magnetic resonance imaging (MRI) for each patient. We have marked the electrical onset on stereoelectroencephalography traces with a blue arrow (A, D, G, J, M, O, Q, T) and the corresponding brain regions are marked by a white arrow (B, E, H, K, N, P, R, U). Brain regions that display fast activity at seizure onset as shown by gamma and IWPR (displayed using red and orange plots over the patients MRI) were included in the resection (C, F, I, L, S, V).

Patient 1 is a 42‐year‐old male with normal intellect. Seizure semiology consisted of sensation of contrast‐enhanced, hyperclear‐vision, loss of consciousness, and genital automatisms. Video‐EEG recordings revealed epileptiform discharges, bilateral frontal predominantly left‐sided involving the mesial and dorsolateral region. Brain MRI was nonlesional. The patient underwent SEEG exploration (Figure 1A) following a left‐frontal hypothesis (Figure 1B). Invasive recordings delineated the epileptogenic zone over the frontal pole, orbitofrontal, anterior cingulate cortex (Figure 1A), which were included in the initial surgical resection. The neuropathological analysis revealed FCDIIA. Seizures relapsed at 6 months postsurgery, and a second cortectomy was performed including the dorsomedial prefrontal, middle cingulate cortex, presupplementary motor area, and superior frontal gyrus (Figure 1C). Genetic testing identified a pathogenic germline mutation in the SZT2 gene. The patient is seizure‐free more than 7 years postsurgery and stopped the anti‐seizure medication (ASM).

Patient 2 is a 7‐year‐old male with normal birth and development. He has focal motor onset seizures starting with a smiling grimace followed by hypermotor behavior. Video‐EEG showed frontal bilateral epileptiform discharges, mostly over the left parasagittal region. Brain MRI was nonlesional. Interictal PET‐CT showed two areas of focal hypometabolism over the left anterior cingulate cortex and frontal operculum. During the SEEG exploration, we recorded multiple habitual seizures that started with an electric pattern consisting in increasing spiking rate followed by low‐voltage fast activity (LVFA) over the anterior cingulate cortex and frontal operculum (Figure 1D,E). At the end of the SEEG exploration, the patient underwent radiofrequency‐thermo‐coagulation (RFTC) of the anterior cingulate, which controlled seizures for 1 year. Based on these data, a cingulate cortectomy was performed with an outcome of 2 years seizure‐freedom postsurgery (Figure 1F). Afterward, rare nondisabling seizures restarted particularly when tapering off medication. Brain tissue analysis revealed FCDIIA. Genetic testing showed a pathologic germline mutation in DEPDC5 gene.

Patient 3 is an 18‐year‐old male with normal birth and intellect. The ictal manifestation consisted in bilateral tonic contraction preceded by a strange sensation in the left hemi‐body particularly the leg. Video‐EEG showed bilateral frontal‐central epileptiform discharges predominantly over the right and the midline leads. The brain MRI was nonlesional. Intracranial recordings showed LVFA starting in the right middle cingulate cortex and spreading over the supplementary motor area and paracentral lobule (Figure 1G,H). RFTC of the middle cingulate was performed and the patient had several months of substantial seizure reduction. He underwent a tailored cortectomy of the right middle cingulate cortex and SMA (Figure 1I), and the histopathology examination concluded FCDIIB. The outcome at 3.5 years postoperatively was worthwhile improvement, monthly seizures instead of multiple per day. The genetic testing identified a NPRL3 pathogenic germline mutation. Subsequently, the patient's brother was diagnosed with nocturnal hypermotor seizures, due to NPRL3 germline mutation.

Patient 4 is a 20‐year‐old male with dyslexia and dysexecutive syndrome. A typical seizure started with fear and a strange sensation over his left hemi‐body particularly in the lower limb followed by hypermotor behavior, bilateral tonic contraction, and face‐flushing. Video‐EEG showed midline and right‐sided frontal‐central abnormalities. The MRI was nonlesional. The SEEG exploration showed LVFA at seizure onset, over the middle cingulate cortex rapidly spreading over the right supplementary motor area, paracentral lobule, anterior cingulate cortex, and anterior insula (Figure 1J,K). Due to eloquent cortex constraints, a cortectomy was performed including the right anterior and middle cingulate cortex and SMA with histopathology showing FCDIIA (Figure 1L). There was an improvement in seizure frequency postoperatively but at the last follow‐up (62 months postsurgery), the patient still had monthly seizures with similar semiology. Genetic testing identified a pathogenic germline mutation in the NPRL3 gene. The patient underwent a second SEEG exploration to explore the relation between the epileptogenic zone, motor, and subgenual cingulate cortex. Two independent seizure onsets were recorded, one over the middle cingulate cortex and paracentral lobule (Figure 1M,N) and another one over the subgenual cingulate cortex (Figure 1O,P). The patient was not considered a surgical candidate, no further therapeutic intervention was done.

Patient 5 is a 20‐year‐old male with normal development. Seizures occurred during sleep and the semiology consisted of limbs paresthesia (predominantly the right side), eye blinking, laughter, hypersalivation, and throat contraction, sometimes bilateralization. Video‐EEG revealed left‐frontal and midline epileptiform discharges. SEEG recordings showed rhythmic spiking and seizure onset with burst of polyspikes and LVFA over the middle cingulate and presupplementary motor area (Figure 1Q,R). The above‐mentioned structures were included in the surgical plan (Figure 1S), pathology showed FCDIIA and the patient is seizure‐free at 4 years postsurgery. He had a particular response to ASM with aggravation under clobazam and substantial seizure control under oxcarbazepine. Genetic testing revealed DEPDC5 germline mutation.

Patient 6 is an 18‐year‐old male with normal birth and development. He has focal motor (left‐side eyes tonic version) to bilateral tonic–clonic seizures. The MRI showed an FCD in the right occipital lobe, resected in another center with no seizure improvement. The second video‐EEG showed right dorsolateral frontal‐central epileptiform discharges. After careful MRI analysis, a second abnormality suggestive of an FCDIIB in the right frontal eye field region was identified. Invasive recordings demonstrated this region to be the seizure onset (Figure 1T,U), and a new surgery was performed including the premotor area, specifically the frontal eye field (Figure 1V). The patient is seizure‐free off medication at 2 years postsurgery. The genetic testing identified pathogenic germline mutation of DEPDC5.

## 4 DISCUSSION

Traditionally, patients with normal brain MRI and epilepsy‐related genetic mutation, particularly ion‐channel gene mutations, were not considered surgical candidates because of wide‐spread involvement of cerebral structures at seizure onset.^11^ In this paper, we describe six cases of drug‐resistant epilepsy due to FCDII and mTOR pathway germline gene mutations. Patients had nonlesional MRI, one initial surgery failure and genetic testing was specifically recommended to decide the surgical strategy, since it was not a routine presurgical investigation.

There is limited experience with epilepsy surgery in GATOR1 gene complex mutations showing favorable surgical outcome in 50%–60% of the cases.^12^ The organization of the epileptogenic network in these types of patients has been reported only in case‐based publications showing bilateral synchronous^13^ or multiple independent epileptogenic zones.^14^ In our case series, patients underwent invasive recordings. They benefited from resective surgery with good outcome: 3/6 seizure‐free, 1/6 only rare seizures, and 2/6 worthwhile seizure reduction, similar to previous reports.^4^ In patients P2, P3, P5, and P6, the EZ had a focal organization in a small sub‐lobar region corresponding to the dysplastic tissue found postsurgically, similar to previously described FCD II MRI‐positive cases.^15^ In P1, we identified a wide‐extended EZ involving the left fronto‐mesial premotor‐prefrontal region. In P4, we found a complex organization of the epileptogenic network, with two SOZ located in different subdivisions of the cingulate cortex (subgenual and middle) that could explain the postsurgical seizure recurrence. The poor outcome in the third case, despite focal organization of the EZ, is related to incomplete resection of the EZ due to eloquent cortex constraints (motor lower limb). Additionally, it seems that NPRL3 mutation‐related epilepsies might have a worst postsurgical outcome (cases 3 and 4 vs. cases 1, 2, 5, and 6). To our knowledge, no second‐hit somatic mutations have been described in NPRL3 cases.

To our knowledge, this case series includes the first reported case of SZT2 germline pathogenic variant in a patient with normal intellect and seizure‐free postsurgery. The SZT2 gene, part of the KICSTOR complex—a lysosome‐associated negative regulator of the mTORC1 signaling^16^ has been previously reported only in infantile‐onset developmental and epileptic encephalopathies.^17^ However, additional data such as somatic second‐hit SZT2 variants and/or other case reports with similar phenotype would be necessary to firmly classify this mutation as clinically relevant. Secondly, our last case presents the first report of two spatially unrelated FCDs (frontal and occipital) in a patient with DEPDC5 germline mutation. Moreover, the occipital FCD was not epileptogenic since its surgical removal did not influence seizure intensity or frequency. It has been previously shown that different epileptogenic lesions or the same lesion type—FCD with a multifocal /bilateral distribution could share the same pathogenic mechanisms^3^ such as timing of mutational event in genes involving the mTOR pathway^18^ or second somatic mutational hit in a different mTOR pathway gene.^19^ We speculate that these could be the mechanisms explaining the two spatially unrelated FCD in P6 even though we lack genetic tissue analysis. Finally, the intracranial seizure onset patterns that we have found in these patients are similar with the ones previously described in FCD^6^ suggesting a common pathophysiological and genetic mechanism of epileptogenesis irrespective of the genetic result—positive or not for the genes that are known up to date to cause FCDs. There are several limitations of this paper; somatic genetic analysis was not available, and family members did not undergo genetic testing.

## 5 CONCLUSION

Intracranial recordings in patients with nonlesional MRI and germline mutations in mTOR pathways could identify a focal organization of the epileptogenic zone and lead to good postsurgical outcome following resective surgery. Intracerebral EEG as confirmation tool of focal and resectable EZ is irrefutable since the extent of the EZ as found by SEEG and its complete removal remain the main predictors for seizure freedom.

## AUTHOR CONTRIBUTIONS

Irina Oane involved in conceptualization, data curation, writing original draft, methodology, validation, review, and editing, Andrei Barborica involved in formal analysis, methodology, software, visualization, review, and editing, Andrei Daneasa, Mihai Dragos Maliia, Jean Ciurea, Sergiu Stoica, Aurelia Dabu, Flavius Bratu, and Camelia Lentoiu involved in data curation, review, and editing. Ioana Mindruta involved in conceptualization, data curation, methodology, project administration, resources, validation, review, and editing.

## FUNDING INFORMATION

This work has been supported by the Romanian, UEFISCDI grant PN‐III‐P4‐ID‐PCE 2020‐0935.

## CONFLICT OF INTEREST STATEMENT

None of the authors has any conflict of interest to disclose.

## Data Availability

The data that support the findings of this study are available on request from the corresponding author. The data is not publicly available due to privacy or ethical restrictions.
